# RESULTS OF SURGICAL TREATMENT OF COLORECTAL CANCER, ELECTIVE AND EMERGENCY, IN PATIENTS WITH COVID-19

**DOI:** 10.1590/0102-672020210002e1644

**Published:** 2022-06-17

**Authors:** Sergio Carlos NAHAS, José Donizeti de MEIRA, Caio Sergio Rizkallah NAHAS, Lucas Faraco SOBRADO, Rodrigo Ambar PINTO, Edson ABDALA, Ulysses RIBEIRO, Ivan CECCONELLO

**Affiliations:** 1 Gastrointestinal and Colorectal Surgery Divisions, Department of Gastroenterology, Hospital das Clínicas, University of São Paulo School of Medicine, São Paulo, Brazil;; 2 Department of Infectious and Parasitic Diseases, University of São Paulo School of Medicine, Cancer Institute of the State of São Paulo, São Paulo, Brazil.

**Keywords:** Covid-19. Coronavirus. Pandemic. Colonic Neoplasms. Colorectal Surgery. Abdomen, Acute., Covid-19, Coronavirus, Pandemia, Neoplasias do Colo.Cirurgia Colorretal, Abdome Agudo.

## INTRODUCTION

The coronavirus disease 2019 (COVID-19) pandemic was identified in Brazil in February 2020. The first Brazilian case was reported on February 25, 2020^4^, and since then, the number of cases increased dramatically, placing Brazil among the countries with the largest number of infected patients and the largest number of deaths from the new coronavirus (>600,000) (4)

A total of 3,000 patients with moderate or severe COVID-19 were admitted to the Hospital de Clinicas of the School of Medicine of the University of São Paulo for in-hospital treatment. Following the recommendations of its infection committee and the main medical societies, all nononcological elective surgeries were suspended.

From 2008 to 2018, the Cancer Institute performed over 8,500 surgeries for colorectal cancer. In this new scenario, although the number of surgeries was reduced to avoid including contaminated patients, some procedures cannot be canceled or postponed; this is especially the case for procedures performed at the Cancer Institute, given the need for continued oncological treatment in both elective and urgent cases. A contingency plan was, therefore, established to allow us to proceed with surgeries that cannot wait due to the risk of disease progression and worsening of the prognosis.

Three patients with colorectal cancer underwent elective (one patient) or urgent (two patients) surgical treatment in April 2020, and they were diagnosed with COVID-19 only during the postoperative period.

## METHODS

This is a retrospective observational study of the first patients diagnosed with COVID-19 during the postoperative period. The patients who were admitted for surgical planning, underwent clinical triage, and did not present any symptoms compatible with COVID-19 at the time of admission were included in the study. Patients signed an informed consent for publication of this series.

## RESULTS

The findings of the surgical treatment of three patients with colorectal cancer who began to show postoperative signs and symptoms of COVID-19 and the changes resulting from the viral process during their postoperative evolution were evaluated.

### Case 1

A 67-year-old male patient presented with a weight loss of 5%, a history of diabetes, and a rectal adenocarcinoma located at 15 cm from the anal margin identified using the magnetic resonance, with extramural vascular invasion and without involvement of the mesorectal fascia - T3bN2. There was no evidence of distant metastasis. The patient underwent conventional rectosigmoidectomy with mechanical end-to-end colorectal anastomosis performed 5 cm below the peritoneal reflection, along with protective ileostomy.

**Figure 1 -:**
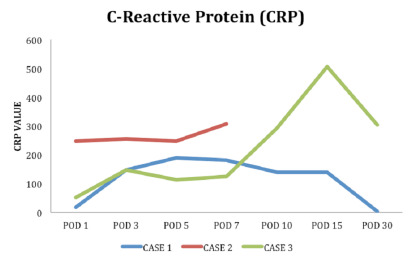
Serum C-reactive protein curves. POD, postoperative day.

**Figure 2 -:**
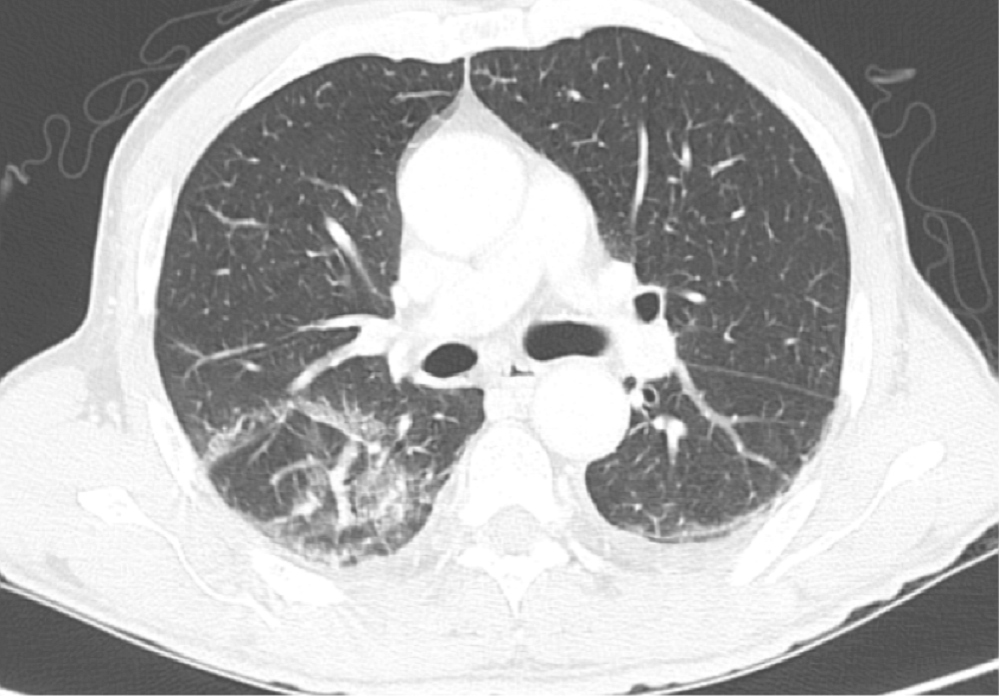
Computed tomography of the chest on postoperative day 4.

Starting on postoperative day (POD) 1, he received enoxaparin for prophylaxis against thromboembolic events. He progressed uneventfully until POD 4, when he presented cough and abdominal pain in the hypogastrium. The patient underwent laboratory tests, which showed an increase in C-reactive protein (CRP) (Figure 1), and a computed tomography (CT) scan of the chest and abdomen showed peripheral ground-glass opacities in the right hemithorax (Figure 2), with no abnormalities in the abdomen. Given the suspicion of COVID-19 based on the clinical and tomographic findings, the patient was placed in isolation. A nose and throat swab was collected to screen for severe acute respiratory syndrome coronavirus-2 (SARS-CoV-2) with real-time polymerase chain reaction (RT-PCR).

Starting on POD 6, peripheral oxygen saturation dropped to near 90%, and the patient required a nasal oxygen catheter at a flow rate of 2 L/min. He was transferred to the Central Institute of the HCFMUSP, which is used exclusively for the care of COVID-19 patients, where he immediately received oseltamivir. After obtaining the positive results for the swab and laboratory data such as a d-dimer level of 6,062 ng/mL and a lactate dehydrogenase (LDH) level of 275 U/L, the patient continued receiving oxygen supplementation by nasal catheter, and treatment for pneumonia was initiated with ceftriaxone^®^ and azithromycin^®^ for 5 days and respiratory physiotherapy.

During the postoperative period day 4, the patient exhibited high output from the ileostomy (>4 L in 24 h), despite the use of maximum doses of loperamide and calcium carbonate, and developed acute renal failure, with an increase in creatinine level of 2.1 mg/dL. At that point, enoxaparin was replaced with unfractionated heparin. Clinical treatment for the recovery of renal function was performed for 10 days, and good response was noticed.

On POD 17, the patient presented with abdominal distension, nausea, vomiting, and reduced intake of food. A CT scan of the abdominal region was performed and showed no mechanical obstruction and gas distension throughout the small intestine until near the ileostomy. Fasting, the use of an open nasogastric tube, and parenteral nutrition were instituted, and remission of the infectious condition caused by COVID-19 was expected. Four days later, the patient showed acceptance of food and intestinal transit. He was retained in the hospital and received nutritional support until his laboratory tests were normal, and he was discharged on POD 30.

### Case 2

A female patient aged 56 years presented with a history of high-grade serous adenocarcinoma of the ovary which had metastasized to the liver, lymph nodes, and peritoneum, underwent chemotherapy, and had a diagnosis of sigmoid adenocarcinoma. She was admitted to the ICESP emergency department due to malignant obstruction being associated with the absence of the release of gas and of bowel movements. A CT scan of the abdomen showed increased volume of peritoneal implants and colonic dilation with fecal content upstream, including in the terminal ileum. First, clinical treatment was proposed, and then fasting, open nasogastric tube, intravenous hydration, correction of fluid and electrolyte imbalances, and antiemetics were prescribed until day 5 of hospitalization without noticing any response.

**Figure 3 -:**
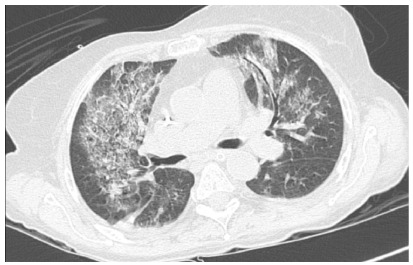
Chest CT of case 2.

Exploratory laparotomy was indicated and found dilatation of small bowel and colon loops, a tumor in the rectosigmoid transition, and peritoneal carcinomatosis causing small bowl loop obstruction. Ileotransverse anastomosis and loop transverse colostomy were performed. The patient presented with adynamic ileus postoperatively, and fasting and nasogastric tube placement were continued. On POD 3, she complained of cough and presented with decreased oxygen saturation requiring a nasal oxygen catheter, high CRP (Figure 1), and chest CT with multiple ground-glass opacities following a COVID-19 pattern (Figure 3). Laboratory assessment showed a d-dimer level of 7,196 ng/mL and LDH of 659 U/L. A nose and throat swab was collected for SARS-CoV-2 screening, which was found positive. The patient progressed with significant clinical worsening and significant alteration of the level of consciousness. Considering the irreversible clinical oncological condition, it was decided to limit life support measures. Death occurred on POD 9.

### Case 3

A 63-year-old female patient presented with an indwelling urinary catheter with fecal discharge. She was recently treated at the emergency department for sepsis of urinary origin caused by an enterovesical fistula in the pelvis. A multidisciplinary surgical approach was proposed. Cystoscopy was performed, and a large tumor mass was found in the bladder. A bilateral ureteral catheter was placed. An exploratory laparotomy was performed, which revealed an unblocked vesicoenteric fistula with contamination of the peritoneal cavity by feces and urine. The patient underwent partial cystectomy, resection of the jejunal segment with enteroanastomosis, and resection of the terminal ileum and cecum with a Mikulicz ileostomy.

On POD 1, she drained 1,500 mL/24 h through a nasogastric tube and 1,000 mL through the ileostomy, and she required vasoactive drugs. Prophylactic enoxaparin was started. Adynamic ileus persisted until POD 5, when parenteral nutrition was started.

**Figure 4 -:**
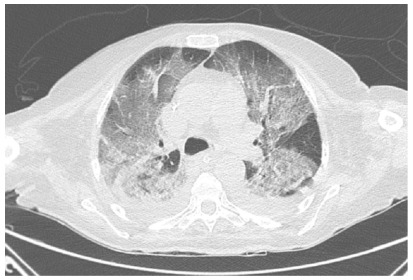
Chest CT of case 3.

On POD 12, the patient showed improved intestinal transit and acceptance of an oral diet but developed a productive cough, dyspnea, desaturation, and severe hemodynamic instability. She underwent another orotracheal intubation and antibiotic therapy with meropenem and vancomycin, started oseltamivir, and required vasoactive drugs (e.g., noradrenaline and vasopressin). Laboratory tests showed d-dimer levels of 26,566 ng/mL, LDH of 673 U/L, and CRP of 448 mg/L (Figure 1), and chest CT (Figure 4) showed diffuse ground-glass opacity across all pulmonary lobes with peripheral predominance, encompassing more than 50% of the lung fields. A nose and throat swab was collected at this time and showed positive for SARS-COV-2.

**Figure 5 -:**
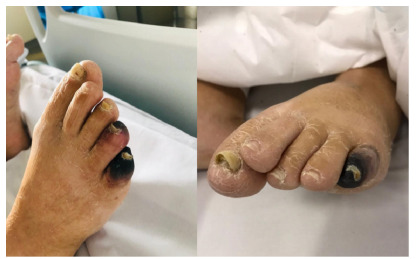
Ischemia of the toes in case 3.

The patient required mechanical ventilation for 12 days. Progressed with a decrease in CRP and d-dimer levels and improvement of ventilatory parameters, the ventilator was removed on POD 24, and the vasoactive drugs were reduced. Ischemic cutaneous lesions were observed in the toes (Figure 5). She exhibited acute renal failure with a creatinine level of 4.0 mg/dL and required continuous hemodialysis. Enoxaparin was replaced with unfractionated heparin. She progressed with improved kidney function but required intermittent dialysis. She continued to exhibit adynamic ileus with a 1-L/24-h flow rate through the nasogastric tube and still required parenteral nutrition. She underwent a CT scan of the abdomen and pelvis on POD 27; the results showed no distension of small bowl loops or points of obstruction but still showed signs of gastric distension. In addition, a small fluid collection was observed adjacent to the bladder, with suspected partial dehiscence of the bladder suture. Antibiotic therapy and antifungal treatment with meropenem, vancomycin, and anidulafungin were initiated.

Starting on POD 30, the patient was transferred to the ward, where she received fluid support to compensate the ileostomy output between 1 and 2 L/day. On POD 32, nasoenteric feeding was introduced, and parenteral nutrition was maintained. She continued to need oxygen therapy to maintain adequate saturation. She presented a worsening of the kidney function, evolving with pulmonary edema and multiple organ dysfunction. Death occurred in POD 48.

The anatomopathological examination of the surgical specimen revealed diffuse large B-cell lymphoma.

## DISCUSSION

The new coronavirus SARS-CoV-2 was first detected in Wuhan (China) and causes COVID-19^17^. The clinical picture of the disease is highly variable, but the most common presentation is characterized by cough, dyspnea, tachypnea, myalgia, asthenia, fever, tachycardia, and rhinorrhea^1^ ^,^ ^6^. In terms of laboratory characteristics, the disease is characterized by lymphocytopenia, increased CRP, and increased LDH^6^.

Its mortality rate ranges from 0.4% to 2.9%^15^ and is even greater for patients who are obese or immunosuppressed, patients with lung or cardiac diseases, elderly patients, and nonsurgical cancer patients^15^, reaching 5.6% in the latter. Mortality is higher in cancer patients because they exhibit greater morbidity and compromised immunity due to the underlying disease ^2^ ^,^ ^15^.

As COVID-19 is a new disease, we do not know all of its possible clinical manifestations. It is known that the most common signs are respiratory ones, but as the disease became pandemic, several other manifestations were described, including a case of intestinal perforation^7^.

In the cases presented, all the manifestations reported in the postoperative period were different from those that are usually observed and reported in relation to COVID-19 in the literature. In all of our patients, after the initiation of intestinal transit and oral refeeding during the postoperative period, there was an abrupt interruption in which all patients had prolonged adynamic ileus and required a nasogastric tube and parenteral nutrition.

As the viral infection improved along with the laboratory and imaging tests, the patients showed return of intestinal transit. The clinical situations observed in these patients and their association with SARS-CoV-2 infection during the postoperative period of colorectal cancer patients were not found in the scientific literature.

Other gastrointestinal symptoms, such as vomiting, diarrhea, and abdominal pain simulating inflammatory acute abdomen, have been described with COVID-19 and may occur before or even in the absence of respiratory symptoms^9^ ^,^ ^10^ ^,^ ^11^. It was observed that the patient in case 1 presented outputs of up to 4 L daily, which may be related to the diarrhea caused by SARS-COV-2. Such high output is rarely observed for such a long time under regular postoperative circumstances.

All patients in this series exhibited acute renal failure during hospitalization, and two of them required dialysis. COVID-19 has been associated with renal failure due to local or systemic processes^5^.

As COVID-19 causes thrombotic microcirculatory events^13^, all patients received subcutaneous anticoagulant therapy. Autopsies of patients who died from COVID-19 have shown thrombosis of the pulmonary parenchymal microvessels^16^, suggesting that the obstruction of the pulmonary capillaries causes a ventilation-perfusion disorder. In addition, there was an improvement in oxygenation in critically ill patients after the administration of anticoagulants in a case series of 27 patients ^8^.

Two of our patients were admitted to the emergency department, and one patient underwent elective surgery. None of the patients in our case series had a clinical or radiological condition compatible with COVID-19 at the time of admission. We understand that the clinical manifestations of COVID-19 during the postoperative period negatively affected the patients’ progression, causing serious complications and prolonging hospitalization. This finding highlights the need for these patients to undergo preoperative testing with RT-PCR of nasopharyngeal and oropharyngeal swabs and be kept isolated from other patients, even when undergoing emergency surgery, to minimize the risks. In the case of elective surgeries, a positive test for COVID-19 should prompt the postponement of the surgical procedure whenever possible. Regarding emergency surgery, preoperative testing helps to institute prevention and control measures in the hospital during the preoperative and postoperative period and to anticipate probable complications, such as acute renal failure or adynamic ileus.

Since this initial phase of patient care during the pandemic at the ICESP-HCFMUSP (March and April 2020), asymptomatic elective patients now undergo clinical screening as well as RT-PCR analysis of nasopharyngeal and oropharyngeal swabs, a specific test for SARS-CoV-2, 48 h before surgery and chest CT scan without contrast on the eve of the surgery, as recommended by several scientific societies^3^ ^,^ ^14^. Urgent patients undergo testing immediately before surgery. Following this protocol allowed safely continuing the surgical treatment of colorectal cancer patients during the pandemic period, as demonstrated in a larger series of our institution^12^.

## CONCLUSION

This series of COVID-19 cases during the postoperative period of colorectal cancer surgery demonstrates that the disease has a significant impact on the postoperative course and high morbidity. The institution of preoperative surveillance, including SARS-CoV-2 testing, can help better determine the best time to perform surgery. The early identification of positive cases minimizes the risks of in-hospital transmission and allows early mitigation of complications from the disease.
